# Comment on Shvedova et al*.* (2016), “gender differences in murine pulmonary responses elicited by cellulose nanocrystals”

**DOI:** 10.1186/s12989-016-0170-4

**Published:** 2016-11-04

**Authors:** Jo Anne Shatkin, Günter Oberdörster

**Affiliations:** 1 Vireo Advisors LLC, Boston, MA USA; 2 University of Rochester, School of Medicine and Dentistry, Rochester, NY USA

**Keywords:** Cellulose nanocrystals, Cellulose nanomaterials, Inhalation, Gender differences, Pulmonary toxicity

## Abstract

A recent publication in “Particle and Fibre Toxicology” reported on the gender differences in pulmonary toxicity from oro-pharyngeal aspiration of a high dose of cellulose nanocrystals. The study is timely given the growing interest in diverse commercial applications of cellulose nanomaterials, and the need for studies addressing pulmonary toxicity. The results from this study are interesting and can be strengthened with a discussion of how differences in the weights of female and male C57BL/6 mice was accounted for. Without such a discussion, the observed differences could be partially explained by the lower body weights of females, resulting in higher doses than males when standardized to body weight or lung volume. Further, few conclusions can be drawn about the pulmonary toxicity of cellulose nanocrystals given the study design: examination of a single high dose of cellulose nanocrystals, administered as a bolus, without positive or negative controls or low dose comparisons, and at an unphysiological and high dose rate. Simulating the bolus type delivery by inhalation would require a highly unrealistic exposure concentration in the g/m^3^ range of extremely short duration. A discussion of these limitations is missing in the paper; further speculative comparisons of cellulose nanocrystals toxicity to asbestos and carbon nanotubes in the abstract are both unwarranted and can be misleading, these materials were neither mentioned in the manuscript, nor evaluated in the study.

## Commentary

In their recent publication, “Gender differences in murine pulmonary responses elicited by cellulose nanocrystals”, Shvedova et al*.,* [1] exposed C57BL/6 mice by pharyngeal aspiration to suspensions of cellulose nanocrystals (CNCs) (40 μg/mouse/day; cumulative dose of 240 μg/mouse). The authors employed a variety of biochemical, cellular, histopathological and physiological measures to compare responses observed in the lungs of male and female mice. As strong advocates for proactive approaches to assessing the safety of nanomaterials, we would be most interested in these findings, however the study design limits the ability to relate the results to effects from CNC exposure under realistic conditions.

The authors state that the “primary goal of this study was to determine whether gender affects pulmonary function, global mRNA expression, and cytokine/chemokine inflammatory responses in the lung of C57BL/6 mice”. The findings of observed gender differences, with females showing a higher pulmonary toxicity is interesting, and as the authors point out, such gender differences in respiratory diseases have been reported in previous studies. However, the observed “gender differences…” would be strengthened with a discussion of how differences in the weights of female and male C57BL/6 mice was accounted for. At 7–8 weeks, when Shvedova et al*.* began their acute exposures, female C57BL/6 mice have an average weight of 18 g while male mice have an average weight of 23 g [2], more than a 20 % difference, which might explain the greater responses in female mice because of the higher dose per unit BW in females. The authors state the C57BL/6 mice used in their study weighed 20.0 ± 1.9 g, but a discussion of the weight distributions of males and females used in their study was not provided, it certainly would strengthen the interpretation of results. The observed gender differences might simply be explained by lower body weights of females, resulting in higher doses than males when standardized with body weight.

As mentioned, the results of the study are difficult to interpret in terms of human health impact, given its overall design. Specifically, the exposure method (pharyngeal aspiration of bolus doses) and examination of effects from a single concentration (240 μg/mouse cumulative exposure), equivalent to a very high deposited dose in humans, requires closer examination. Shvedova et al*.* estimated that the CNC dose administered to mice is equivalent to a human worker exposed to the Occupational Safety & Health Administration (OSHA) limit for 42 days. However, it is scientifically not justifiable in terms of effects to equate a deposited bolus dose (exposure duration is a fraction of a minute) with the same dose achieved after an exposure for many days in the lung. A more realistic comparison of the dose in mice to the dose deposited in workers’ lungs has to consider the following: Key is that there is a difference in effects and underlying mechanisms induced by very high *vs*. very low dose rates [3]. Effects induced by high bolus-type delivery can be used for hazard identification, provided that dose–response data are established to determine a slope [4]. Unfortunately, though, results from such studies cannot be used for risk characterization (establishing limit values).

The principle of our approach for mouse-human extrapolation modeling involves the following steps: We used the Multiple-Path Particle Dosimetry (MPPD) model (Version 3.04) to determine the deposited fraction inhaled by a 20 g mouse of an aerosol with mass median aerodynamic diameter (MMAD) of 0.6 μm and geometric standard deviation (GSD) of 2.0 and aerosol density of 1. Respiratory parameters (tidal volume and breathing frequency) were allometrically adjusted to body weight which is essential when running the MPPD model. The model derived deposition fraction in the alveolar region of the mouse lung gave a value of 5.1 %.

As a next step, an estimate of the deposited dose in humans over an 8 h (hr) workplace exposure was performed, at a concentration of 5 mg/m^3^, which is the OSHA occupational limit for respirable cellulose dust. The deposition fraction in the alveolar region of the human lung, using the MPPD model with the MMAD and GSD given above, turned out to be 8.1 %. The 8-h deposited dose in the alveolar region under light physical exercise breathing conditions was calculated as 3,985 μg/day at 5 mg/m^3^ exposure concentration. This is equivalent to 6.3 ng/cm^2^ of the alveolar surface area (634,620 cm^2^ at functional residual capacity [FRC]) in the human lung. The equivalent deposited dose in the mouse by inhalation would then be 3.3 μg/mouse for a one day (8 h) exposure (mouse alveolar surface area at FRC of 526 cm^2^ x 6.3 ng/cm^2^). This is 12 times less than the 40 μg/mouse delivered in the study. In addition, as mentioned above, the impact of an 8-h inhalation exposure *vs*. a less than a minute bolus delivery has to be considered.

Finally, in order to determine a mouse equivalent inhalation exposure concentration that results in the same deposited lung dose as human workers deposit when exposed to the OSHA limit of 5 mg/m^3^ for 8 h, we used the following correlation:

Deposited Dose = Minute Ventilation x Expos. Concentration x Depos. Fraction x Expos. Duration

(The deposited dose over 8 h is 3.3 μg/mouse; the MPPD derived deposition fraction is 0.051 [see above]; body weight allometrically adjusted tidal volume and breathing frequency for a 20 g mouse are 0.148 ml and 252/min, respectively; and exposure duration is 8 h). Rearranging and solving the above equation for Exposure Concentration gives a value of 3.6 mg/m^3^, which is in a similar range as the OSHA exposure limit for workers.

However to simulate the bolus type delivery used in the mouse study, the dose of 3.3 μg/mouse would have to be inhaled in one minute - rather than 8 h - at an exposure concentration of 1.73 g/m^3^. Nobody would claim this Exposure Concentration to be realistic; and yet, that is exactly the equivalent to bolus-type dosing in terms of the dose delivered to the respiratory tract. Unfortunately, this has been done - and continues to be done, and accepted without question - in numerous other studies. (Additional issues of unequal distribution between aspiration and inhalation are not considered here).

Conclusions from this derivation of a human/mouse equivalent dose in the alveolar region are: (*i*) Shvedova et al*.* exceeded the estimated human daily deposited dose—at the Permissible Exposure Limit allowable by OSHA - by a factor of 12 when dosing the mice. (*ii*) simulating the bolus type delivery with inhalation would require a highly unrealistic exposure concentration in the g/m^3^ range of extremely short duration; (*iii*) effects and induction of underlying mechanisms are due to the high, unrealistic and unphysiological exposure conditions which have to be interpreted with great caution [5]. Part of any study must be a critical assessment of the relevance of administered doses in animal (and in vitro) experimental studies in order to avoid erroneous conclusions. For example, the reported significant gender differences in this study may be simply a result of differences in male/female body size if doses have not been adjusted; or are they due to the study design of only one very high dose? Again, determining the slope of a dose–response relationship would be essential to answer these and other questions. We encourage further discussion of the importance of dosing, which would include more details of our dosimetric calculations.

In order to comment on the possible human toxicity of CNCs, the authors should have investigated several doses in order to demonstrate response related to dose of CNC exposure. This is especially important given that high-dose effects in in vivo studies are inherently difficult to interpret. It is well documented that dose-dependent transitions in the principle mechanism of toxicity occur at high exposures [5]; for example, high doses - amplified by very high dose rates - may result in non-linearity of responses, effects occurring from saturated receptor pathways (for both activating and detoxifying interactions), and inflammation due to conditions of overwhelming defenses [5] that are not representative of effects from realistic dose exposures.

The lack of negative and positive controls of known agents, together with the lack of different doses to characterize dose–response relationships, limits the ability to conclude there are substance-specific effects rather than as result of inflammation due to simple foreign particle introduction resulting from a bolus, high-dose exposure; similar outcomes are known to occur from high doses of any poorly soluble dust. The study design incorporates interesting measures of gene and protein expression following exposure, however the issue is not discussed regarding whether these high dose responses relate specifically to CNC, or might similarly occur due to common respiratory triggers (such as other fibrous and non-fibrous particle types) at these exposure levels. The lack of low dose testing similarly limits interpretation of the gene and cytokine responses.

We question, further, why the abstract includes an unexpected statement comparing CNC to both carbon nanotubes and asbestos, never to be mentioned again in the paper. A comparison to asbestos as a positive benchmark control was not part of the study design, neither was a negative benchmark included. Adding such benchmarks - combined with a dose–response approach - would have enhanced the study beyond a simple design by allowing to rank CNC against well-characterized benchmarks. Without this, referring to asbestos solely in the abstract is unwarranted and unjustified.

The sulfated CNCs examined by Shvedova et al*.* were 158 nm long and are not classifiable as World Health Organization (WHO) fibers [6]. Additionally, they are 1–2 orders of magnitude shorter than fibers we would expect to induce mechanisms that lead to toxicity under the asbestos fiber toxicity paradigm, where fibers longer than 15–20 μm are critical [7]. We are not suggesting that inhalation of high doses of CNCs would not cause the inflammatory response observed by Shvedova et al., however the toxicity observed is better expressed in terms of the very high lung burden, rather than comparing it in the abstract to asbestos.

In summary, readers of “Particle and Fibre Toxicology” should recognize that the study outlined by Shvedova et al*.* was an investigation into gender differences of pulmonary toxicity from bolus-type lung exposure at a high dose of a poorly soluble dust. The conclusions that can be drawn about the pulmonary toxicity of CNCs - that very high doses of CNCs cause inflammation - are common to even benign fibrous and non-fibrous particles.

## Abbreviations

CNC: Cellulose nanocrystals
FRC: Functional residual capacity
GSD: Geometric standard deviation
MMAD: Mass median aerodynamic diameter
MPPD: Multiple-Path Particle Dosimetry
OSHA: Occupational Safety & Health Administration
WHO: World Health Organization
