# Supplementary material for: Comment on Shvedova et al. (2016), “gender differences in murine pulmonary responses elicited by cellulose nanocrystals”
Source: Part Fibre Toxicol. 2016 Nov 4;13:59. doi: 10.1186/s12989-016-0170-4 (PMC5096324; doi:10.1186/s12989-016-0170-4)
Supplement: Related texts

# Reply: Authors’ response to: comment on Shvedova et al. (2016), “gender differences in murine pulmonary responses elicited by cellulose nanocrystals”

**Authors:** Anna A. Shvedova, Elena R. Kisin, Naveena Yanamala, Mariana T. Farcas, Autumn L. Menas, Andrew Williams, Philip M. Fournier, Jeffrey S. Reynolds, Dmitriy W. Gutkin, Alexander Star, Richard S. Reiner, Sabina Halappanavar, Valerian E. Kagan

**Affiliations:** 3 Exposure Assessment Branch, NIOSH/CDC, Morgantown, WV USA; 4 Engineering and Controls Technology Branch, NIOSH/CDC, Morgantown, WV USA; 5 Department of Physiology & Pharmacology, School of Medicine/WVU, Morgantown, WV USA; 6 Environmental and Radiation Health Sciences Directorate, Health Canada, Ottawa, Ontario K1A 0K9 Canada; 7 Department of Chemistry, University of Pittsburgh, Pittsburgh, PA USA; 8 Department Pathology, University of Pittsburgh, Pittsburgh, PA USA; 9 Forest Product Laboratory, USDA Forest Service, Madison, WI USA; 10 Free Radical Center, University of Pittsburgh, Pittsburgh, USA; 11 Department of Environmental & Occupational Health, University of Pittsburgh, Pittsburgh, PA USA

We would like to thank Drs. Shatkin and Oberdörster for their interest and comments on our paper [1] and we would like to take this opportunity to clarify some of the issues raised. One of the specific comments is focused on the relevance of our original findings to differences in the total body weights between the female and male mice. Our study determined the respiratory toxicological endpoints after several pharyngeal aspiration exposures to cellulose nanocrystals (CNC) in male and female mice with a specific focus on the comparison of the relative responses associated with gender differences. Significant differences in the responses to respirable CNC with a higher pulmonary toxicity in female mice were described. However, Shatkin and Oberdörster suggest that these differences might be due to the lower body weights of female mice, resulting in higher relative doses vs males. Accumulating evidence suggests that gender can have a profound effect on incidence and severity of a variety of pulmonary diseases [8–10]. It is well known that changes in the lung volume/mass, rather than body weight, define the respiratory abnormalities. Additionally, it has been established that the lung volume/mass of male and female mice of the same age are not different, in spite of the differences in the body weights [11, 12]. As per Environmental Health Criteria 239 [13], the tissue dose which is the amount distributed to and present in a specific tissue of interest, in this case – the lung, would be in fact, the same for male and female mice. In our study, only the *pulmonary* responses were compared between male and female mice: inflammation and damage, TGF-β, and collagen, oxidative stress and pulmonary functions as well as the global mRNA expression were measured in lung. Thus, the comment on the employed doses with respect to the total weight differences between males and females is without merit. We maintain that the stronger responses to CNC documented in female mice were due to the gender associated differences in the pulmonary reactivity, rather than to 14 % variance in the total body weight between the female and male mice. We thank Shatkin and Oberdörster for raising this issue in their letter [14], and the opportunity to expand our discussions. Further the comment by Shatkin and Oberdörster was useful as it allowed us to explain an important point: that in spite of the slight differences in the total body weight between female and male mice – there was essentially no gender differences between the lungs of the animals either in terms of their weight/area or functions.

Shatkin and Oberdörster expressed concerns that the dosages employed in this study exceeded the estimated human daily deposited dose – at the Permissible Exposure Limit allowable by OSHA - by a factor of 12. It has to be acknowledged that direct quantitative comparisons between rodent and human toxicological assessments are difficult to make. This is due to uncertainties associated with various methods/tools available for modeling nanomaterial deposition in the lungs and/or differences in the physicochemical characteristics inherent to each material being investigated, as well as those related to each species. With full understanding of these limitations, the dose responses in mice can still provide useful information for meaningful modeling and approximate evaluations relevant to realistic human exposure scenarios. Several mathematical models, including the MPPD method preferred by Shatkin and Oberdörster, have been developed and used by various groups to improve translation of the in vivo rodent assessments to corresponding human equivalent exposures [15–18]. In our study, mice were exposed over an 18 day period to 6 single doses (once every 3 days) of 40 μg of CNC materials using a pharyngeal aspiration technique (cumulative dose ~240 μg/mouse) in lieu of a single bolus dose. Therefore, for estimating human equivalent exposures to attain similar lung burdens, we opted to make several assumptions including no clearance over the 18 day period and the equivalency of the exposure dose to the dose deposited in the alveolar region. In our comparisons, we first estimated lung burden in rodent models, then normalized it to lung burden/alveolar epithelial surface area to further estimate human equivalent exposure time period as follows.

Human Alveolar Deposition = Exposure Concentration x Ventilation/8 h working x Deposition Fraction

- OSHA Conc. of allowable exposure to Cellulose = 5 mg/m^3^ (respirable fraction)
- Ventilation / 8 h working (20 L/min * 0.001 m^3^/L * 60 min/h * 8 h/day) = 9.6 m^3^/d ([19])
- Deposition fraction for CNC having largest dimension ~100-300 nm [13] = ~15 % ([19, 20])
- Total alveolar surface area of human lung = 634,620 cm^2^ at functional residual capacity [FRC]
- Mouse alveolar surface area = 526 cm^2^ at FRC.

Based on this, the lung burden in humans per day can be estimated as 7200 μg or 11.4 ng/cm^2^ per day. Thus, the accumulated alveolar lung burden in humans ignoring the clearance and other potential factors over 18 days will be 18*11.4 = ~205 ng/cm^2^. The T_1/2_ for alveolar clearance in humans is ~ 1 year and can be ignored in these estimates, as the clearance would be insignificant over the 18 days required to achieve the equivalent worker lung burden. Assuming that the CNC particles administered through pharyngeal aspiration deposit predominantly in the deep lungs (the alveolar region), the dose employed in mice would result in a lung burden of ~0.456 μg/cm^2^ or 456 ng/cm^2^, i.e. only ~ 2.3 times higher than can be expected from equivalent exposures per day in humans. Thus we estimated that the human equivalent lung burden would be achieved in ~42 days (2.3 x 18 days = 41.4 days). It has to be noted that if the total dust concentrations, instead of respirable fraction, of OSHA Permissible Exposure Limit (PEL – 15 mg/m^3^) or NIOSH Recommended Exposure Limit (REL – 10 mg/m^3^) were considered, then the respective human alveolar lung burden would be equivalent to 34 ng/cm^2^ or 23 ng/cm^2^, respectively. Over 18 days, this would result in the accumulation of 612 or 414 ng/cm^2^, which are slightly higher than or closer to the average CNC dose in mice per day employed in our study. Moreover, the employed exposure regimen and concentrations over 3-weeks of exposure are further justified as one considers that humans may be exposed chronically for longer periods of times.

Can the effects reported in our study be due to non-linear responses stemming from receptor oversaturation pathways and/or conditions of overwhelming the defenses—lung overload phenomenon? So far, this has been demonstrated in rats, but not in mice or humans. Porter et al. [21, 22] demonstrated that a lung burden of 6 mg/rat of exposure to silica particles (alveolar surface area ~0.4 m^2^ of rat lung [23, 24] had not reached overload and had not decreased the clearance rate. This is equivalent to 0.9 mg/mouse lung (alveolar surface area ~0.06 m^2^ of mouse lung [23, 24]) which is ~4 times higher than the concentrations of CNC (up to 240 μg/mouse) we have investigated in several of our studies [1, 25, 26]. Importantly, several studies indicated that the dose dependent effects of nanomaterials were mostly linear within this dose range.[25].

Shatkin and Oberdörster overlooked one of the most essential experimental features of our study [1]: the employment of chronic treatments achieved by scheduled repeated exposures (twice a week for 3 weeks resulting in an accumulated dose of 240 μg) to deliver CNC by pharyngeal aspiration. This regimen achieves the accumulation of particles in the lung over time, which is closer to potential occupational exposures than a single daily bolus dose (as supposed by Shatkin and Oberdörster). Importantly, this protocol may be one of the best ways to dose animals in cases when generation of nanomaterial aerosol for inhalation studies (eg, nano-cellulose materials) could represent technical difficulties [27]. Several studies have documented the noninvasive and reproducible character of particle deposition and clearance from the mouse lower respiratory tract after pharyngeal aspiration [28, 29]. Moreover, direct comparisons of bolus inhalation vs aspiration exposures of SWCNTs further demonstrated the efficiency of the aspiration technique in studies of fibrous particles [30]. The repeated exposure regimens have been employed by others for pharyngeal aspiration [18, 31–33] and intra-tracheal instillation exposures [34–37]. Furthermore, the deposition estimates using MPPD model (v3.0) preferred by Shatkin and Oberdörster also have several limitations. These include (a) deposition models for mouse are based on only two strains (BALBc and B6C3F1)—both of which are models for either asthmatic or polygenic diseases, (b) calculations on mouse extrapolation are based on experimental data for spherical particles, whereas CNC particles with elongated structures could exhibit different kinetics, (c) generally low estimates of alveolar surface area (64.5 m^2^ vs 102 m^2^ for humans and 0.03 m^2^ vs 0.06 m^2^ for mice), and (d) consideration of endotracheal vs nose-only exposure.

Why did we choose a cumulative dose of 240 μg/mouse? We agree that determining the slope of a dose–response relationship is important for the assessment of the relevance of the mouse doses used in our study to human exposures. The dose response of bolus adverse effects of CNC exposure (50 – 200 μg/mouse) in mice by pharyngeal route has been published previously [25]. Our study described dose–dependent effects assessed by several outcomes including inflammation, cytokine/chemokine release, pulmonary damage, and oxidative stress markers - protein carbonyls and 4-hydroxynonenal. Accordingly, the doses selected for repeated exposure regimen in this study were similar to our low-dose bolus exposure (40 μg/mouse) given to mice twice a week for 3 weeks thus reaching cumulative dose of 240 μg/mouse closer to the highest CNC bolus dose.

Shatkin and Oberdörster [14] further commented on the lack of positive and/or negative controls in our study. Our previous work demonstrated that asbestos administration (employed as a positive control) at the same lowest CNC dose (50 μg/mouse) demonstrated lower acute toxicity compared to CNC particles at equal mass concentration [38]. While detailed comparisons of pulmonary toxicity and long term effects of cellulose nanocrystals with asbestos and other fibrous materials (e.g., CNTs, CNF…) are definitely of great interest, they were not the topic of this study and we are not suggesting from our present data that CNC are asbestos-like with respect to the spectrum of asbestos induced diseases. We share the common opinion that such studies, together with the comparison of inhalation studies, are essential for the further assessment of possible human toxicity of CNCs.

Finally, Shatkin and Oberdörster [14] question whether comparisons of CNC to carbon nanotubes and asbestos was necessary in the abstract. It is common knowledge in the field of nanotoxicology that high aspect ratio materials, particularly carbon nanotubes, can be potentially pathogenic like asbestos. The similarity in mechanisms and pathways of toxicity have been articulated and emphasized in many published papers and included in their titles or abstracts [39–46] as well as in studies of nanocellulose materials [25, 47–49]. Thus, while not directly investigated in the current study, this paradigm has been widely accepted by the toxicology community, it was not directly investigated in the current study and we agree that this inference should have been left out.

In summary, we do not believe that the slight differences in the total body weights of female and male C57BL/6 mice contributed to the elevated responses in female mice upon exposure to CNC. Our study was aimed at the investigation of gender differences of pulmonary toxicity from repeated pharyngeal aspiration exposures to CNC materials in mice, rather than exploration of dose–response effects of CNCs. We believe this study representing a “proof of principle” or “hypothesis forming” study [27], was an investigation into gender differences of pulmonary toxicity from repeated pulmonary aspiration exposure to CNC and needs to be followed up by long term inhalation studies, as rightfully emphasized by Shatkin and Oberdörster [14]. Further, the specific gene expression changes related to carbohydrate/pattern /polysaccharide and glycosaminoglycan binding and signaling, as detailed in our paper, support a biological response as a result of CNC exposure. Taken together, our data provide evidence that raise doubts concerning the validity of the conclusion drawn by Shatkin and Oberdörster [14] that very high doses of CNCs cause inflammation and that such pulmonary responses are common to even benign fibrous and non-fibrous particles. However, we do agree that there is a critical need for further detailed research aimed at mechanistic understanding of potential risks of human exposure to CNCs. Studies in our research group, detailing the pulmonary effects of CNCs via inhalation exposure route at relevant exposure limits, are currently underway. Finally, we would like to thank Shatkin and Oberdörster for their thought provoking commentary [14] highlighting the critical need for continuous research aimed at better understanding of the potential significance and risk for human exposures to CNCs.
